# A systematic approach toward progressive improvement of national antimicrobial resistance surveillance systems in food and agriculture sectors

**DOI:** 10.3389/fvets.2022.1057040

**Published:** 2023-02-07

**Authors:** Nicolas Keck, Michaël Treilles, Mary Gordoncillo, Ouoba Labia Irène Ivette, Gwenaëlle Dauphin, Alejandro Dorado-Garcia, Suzanne Eckford, Emmanuel Kabali, Morgane Gourlaouen, Francesca Latronico, Juan Lubroth, Keith Sumption, Junxia Song, Béatrice Mouillé

**Affiliations:** ^1^Food and Agriculture Organization of the United Nations (FAO) Headquarters, Rome, Italy; ^2^Emergency Centre for Transboundary Animal Diseases (ECTAD), Regional Office for Asia and the Pacific, Food and Agriculture Organization of the United Nations (FAO), Bangkok, Thailand; ^3^Regional Office for Sub-Saharan Africa, Food and Agriculture Organization of the United Nations (FAO), Accra, Ghana

**Keywords:** FAO-ATLASS, antimicrobial resistance, surveillance, laboratory, assessment, food, agriculture, One Health

## Abstract

The first Food and Agriculture Organization of the United Nations (FAO) Action Plan on antimicrobial resistance (AMR), published in 2016, identified the need to develop capacity for AMR surveillance and monitoring in food and agriculture sectors. As part of this effort, FAO has developed the “Assessment Tool for Laboratories and AMR Surveillance Systems” (FAO-ATLASS) to assist countries in systematically assessing their AMR surveillance system in food and agriculture. FAO-ATLASS includes two different modules for surveillance and laboratory assessment. Each module includes two questionnaires that collect either qualitative or semi-quantitative data to describe and score the performance of national AMR surveillance system data production network, data collection and analysis, governance, communication and overall sustainability in a standardized manner. Based on information captured in the questionnaire by trained assessors (1) tables and figures describing the outputs of the surveillance system are automatically generated (2) a Progressive Improvement Pathway (PIP) stage, ranging from “1-limited” to “5-sustainable”, is assigned to each laboratory assessed in the country, each area of the surveillance system and also to the overarching national AMR surveillance system. FAO-ATLASS allows national authorities to implement a strategic stepwise approach to improving their AMR surveillance systems *via* the FAO-ATLASS PIP system and provides an evidence base for actions and advocacy. The implementation of FAO-ATLASS at regional and global levels can contribute to harmonize and better coordinate strategies aimed at implementing an integrated AMR surveillance system under the One Health approach.

## Introduction

Antimicrobial resistance (AMR) is a major global human health concern causing potential increase in treatment failures, loss of treatment options and increased likelihood and severity of infectious disease. A recent publication on the global burden of antimicrobial resistance studying health consequences attributable to bacterial AMR for 23 pathogens in 204 countries and territories in 2019 estimated that 1.27 million deaths were directly attributed to resistance in 88 pathogen-drug combinations evaluated (1). Besides human health, AMR is also a concern for animal health and can consequently have serious impact by limiting the possibilities of treatment or increasing the treatment costs in agriculture and animal productions. In these sectors, the global consumption of antibiotics will likely increase in the future because of the growth in consumer demand for livestock products in middle-income countries and a shift to large-scale farms (2). The risk due to AMR appear particularly high in countries where legislation, consumer pressure, surveillance systems, and the prevention and control of infectious diseases are weak or inadequate.

The current AMR crisis can only be addressed by adopting a One Health approach globally, meaning that veterinary medicine, agriculture, and environment sectors will play key roles in cooperation with the human health sector. In that context the World Health Organization (WHO) adopted during its 68th Assembly in May 2015 a Global Action Plan to combat AMR (3), highlighting the need to address the AMR crisis using One Health approach with the involvement of human health and veterinary authorities, food and agriculture sectors, financial planners, environmental specialists, and consumers. FAO's Thirty-ninth Conference adopted Resolution 4/2015 in June 2015, and published two consecutive FAO Action Plans on AMR to support the Global Action Plan (4, 5). The FAO action plan on AMR 2021–2025 addresses five major focus areas in food and agriculture sectors: (1) Increasing stakeholder awareness and engagement to foster change, (2) Strengthening surveillance and research to support evidence-based decisions, (3) Enabling good practices to prevent infections and control the spread of resistant microbes, (4) Promoting responsible use to keep antimicrobials working and (5) Strengthening governance and allocating resources to accelerate and sustain progress.

Among these areas, AMR surveillance is the cornerstone for assessing and monitoring the emergence and the spread of AMR and for providing evidence for action. A sound surveillance system implemented for continuous monitoring of AMR helps to reduce and control AMR and Antimicrobial use (AMU) by providing information for targeted regulation, advocacy, awareness raising, and tailored interventions to address the development and transmission of resistance. At the global level, AMR surveillance provides early warning of emerging threats and data to identify long-term trends. At the national level it guides policy makers and helps them to apply appropriate and timely interventions. At the local level it allows actors in the field (veterinarians, para-veterinarians, farmers, crop producers) to take better decisions for the treatment of animal and plant infectious diseases. To achieve these goals, an AMR surveillance system must generate up-to-date, comparable, representative, high quality data on pathogens or indicators of concern from the target populations.

Several challenges for the establishment of AMR surveillance networks have been particularly identified in low- and middle-income countries, especially because AMR surveillance in the animal sector is still in its infancy (6). On human health side, WHO developed assessment tools for laboratories (7) as well as a checklist for AMR surveillance system (8). On the Food and Agriculture side, FAO developed an Assessment Tool for Laboratories and Antimicrobial Resistance Surveillance Systems (FAO-ATLASS) to support countries in strengthening the generation of high quality AMR data for evidence. This paper presents the structure of the tool, the methodology of the assessment and the outputs and recommendations of a national assessment using FAO-ATLASS.

## Scope and development of FAO-ATLASS

FAO-ATLASS aims to assist countries in systematically assessing their AMR surveillance systems in the food and agriculture sectors by (1) mapping laboratory networks and activities to detect AMR, as well as the national AMR surveillance system (2) measuring in a standardized way the capacities and capabilities of laboratories and the AMR surveillance activities. FAO-ATLASS was developed in alignment with international standards set by the World Organization for Animal Health (WOAH), and the Codex Alimentarius Commission (CAC) as the body responsible for all matters regarding the implementation of the Joint FAO/WHO Food Standards Programme (9, 10).

The tool is currently focused on surveillance of antibiotic resistance in bacteria isolated from animals, food and feed products, plants and environment samples, sources that are considered highest risk, both from human health and animal health point of view. In FAO-ATLASS and in this article, Food and Agriculture sectors include terrestrial and aquatic animal health, food and feed safety, plant health and environment.

Although some information on AMU and residues surveillance are included in FAO-ATLASS to provide basic information, the tool is currently focused on AMR surveillance and associated laboratory activities in bacteriology. Antimicrobial use, antimicrobial residues in food products, and AMR are linked and should be evaluated in a complementary way by different methods.

To ensure consistency with the major assessment tools used at the international level, the development of FAO-ATLASS was built on existing tools and materials:

- The FAO tool dedicated to the evaluation of national surveillance in animal health (11)–FAO-SET–by selecting and adapting some questions relevant to AMR surveillance,
- The FAO Laboratory Mapping Tool (12)–FAO-LMT–for questions related to laboratory functionality, completed with some specific questions regarding bacterial identification, and AMR detection,
- The Joint External Evaluation tool, especially the specific chapter on the prevention of AMR (13),
- The questionnaire from the tripartite AMR country self-assessment survey (14),
- International guidelines for the implementation of an AMR surveillance system (15–18),
- International technical guidelines or standards on antimicrobial susceptibility testing, especially from the European Committee on Antimicrobial Susceptibility Testing and Clinical and Laboratory Standards Institute,
- Opinion from experts in the field of antimicrobial susceptibility testing and AMR surveillance.

A FAO expert team developed the first version of the tool in 2015. The tool was then reviewed in two rounds of consultations (2016 and 2019) by more than 20 reviewers with multidisciplinary expertise (epidemiology, public health, laboratory management, policy measures and development) and working in different national agencies as well as international organizations (WOAH and WHO). These reviews consisted in a qualitative assessment of the tool to check exhaustively terminology, feasibility, and agreement with other assessment tools and available AMR surveillance standards. All comments were considered and discussed with the reviewers when necessary.

Between the two rounds of consultations, the first pilots in country testing were conducted in 2016 in Senegal and Kenya, then followed by several missions in Asia and Africa in 2017. The outcomes of those reviews, in-country pilot tests and feedback from users after missions, were used to progressively refine the tool to obtain a finalized version in 2021.

## Structure of FAO-ATLASS

The tool is divided into two modules (two different Microsoft Excel^®^ files), each consisting of a descriptive questionnaire and a semi-quantitative questionnaire:

1) The surveillance module which requires answers from respondents working in different institutions involved or supposed to be included in the surveillance system and is completed at nationwide level. Questions cover five main areas of the AMR surveillance system: governance, data production network (laboratories), data collection and analysis, communication, and sustainability. This module is composed of two questionnaires:

- “Surv”: The descriptive questionnaire is composed of 85 questions organized into the five main areas of the AMR surveillance system and nine categories. Besides those questions focusing specifically on AMR, 15 ancillary questions concern the collection of basic information of the surveillance for antimicrobial use and antimicrobial residues. This questionnaire depicts the organization and outputs of the surveillance system: general national multi-sectoral framework, linkages with human health, actors involved and their roles, modalities of the AMR surveillance implementation in the different sectors (including the sample types, methods for AMR detection, indicators under surveillance, and AMR testing funding), organization of the laboratory network on AMR, upstream and downstream communication, sustainability and continuous improvement.
- “SET-AMR” (Surveillance Evaluation Tool for AMR): The semi-quantitative questionnaire is composed of 36 questions organized according to the five areas of the AMR surveillance system (Table 1).

2) The laboratory module is completed individually for each laboratory assessed. The number of assessed laboratories may vary among countries. Laboratories to be assessed are those included, or intended to be, in the AMR national surveillance system for the food and agriculture sectors. The assessment of the laboratories covers four areas: activity, technical practices, management of data and biological material, and quality assurance. These laboratory assessments can be considered as complementary to a normative evaluation, for example according to ISO 17025 standard. Indeed they tackle in a very broad (e.g., antibiotics which are tested, modalities of data and biological storage etc.) the organization of laboratories with a view to their participation in an AMR surveillance system. This module is composed of two questionnaires:

- “Lab”: The descriptive questionnaire is composed of 70 questions organized into the four main areas and 16 categories. This questionnaire depicts the activity of the laboratory in the field of bacteriology and antimicrobial susceptibility testing: number of samples tested, resources, technical practices in bacteriology (isolation, identification) and antimicrobial susceptibility testing (methods used, antibiotics tested, standards for results interpretation), management of data and biological material, quality assurance (use of Standard Operating Procedures, use of reference strains, participation to proficiency testing).
- “LMT-AMR” (Laboratory Mapping Tool for AMR): The semi-quantitative questionnaire is composed of 42 questions organized in 12 categories (Table 2). Based on the LMT-AMR, a specific sheet (LMT-BACT: 31 questions) has been developed to assess the laboratories which are not conducting antimicrobial susceptibility testing but are conducting bacterial isolation and providing isolates to the network for AMR surveillance purpose.

**Table 1:** Information collected in FAO-ATLASS SET-AMR questionnaire.

**Area**	**Subcategory**
Governance	Existence of an operational structure representative of the stakeholders involved in AMR surveillance under One Health approach (multi-sectoral working group(s) or coordination committee on AMR)
	Development of a National Action Plan on AMR involving the food and agriculture sectors
	Relevance of AMR surveillance objectives and AMR indicators in food and agriculture sectors
	Regulations on AMR surveillance organization in the food and agriculture sectors
Data collection and analysis	Existence of an operational management structure (central epidemiology unit) in food and agriculture sectors
	Frequency of coordination meetings between central epidemiology unit with local units
	Representativeness of the surveillance sampling scheme in food and agriculture sectors including environment
	Adequate skill level in AMR epidemiology of members of the central unit
	Adequacy of the data management system for the needs of the AMR surveillance system (database, etc.)
	Data input interval in accordance with the objectives and use of AMR surveillance system results
	AMR data verification and validation procedures formalized and operational
	Analysis of AMR data against system requirements
Data production network	Effective integration of competent laboratories in the AMR surveillance system
	Level of the standardization of work between different laboratories involved in the AMR surveillance system
	Relevance of laboratory diagnostic techniques
	Technical level of AMR data management of the laboratory network
	Frequency of data transmission to the epidemiology unit
	Harmonization of data transmitted to the epidemiology unit
Communication	External policy for communication with decision makers and other stakeholders
	Identification and coverage of key stakeholders' expectations about the results of the surveillance system
	Existence of awareness building AMR programs for surveillance actors
	Communication of risk assessment outcomes to relevant parties
	Regular release of reports on AMR surveillance results
	Systematic distribution of AMR surveillance results to field actors (outside of a report)
	Presence of a communication system organized between field actors (mail, websites, telephone…)
Sustainability	Adequacy of material and financial resources for the multi-sectoral working group(s) or coordination committee on AMR
	Adequacy of financial resources for the implementation of the National AMR action plan
	Adequacy of human, material, and financial resources for AMR data production (laboratory network) needs
	Adequacy of human, material, and financial resources for AMR data collection and analysis (epidemiology) needs
	Adequacy of human, material, and financial resources for communication needs
	Regular advanced training for actors of the surveillance
	Adequacy of material and financial resources for training
	Development and validation of performance indicators for AMR surveillance system
	Regular measurement, interpretation, and dissemination of performance indicators
	External assessment carried out
	Implementation of corrective measures

**Table 2:** Information collected in FAO-ATLASS LMT-AMR questionnaire.

**Area**	**Category**	**Subcategory**
Activity	Sustainability	Financial capacity (allocation of funds)
		Management
	Workflow organization	Quality of samples submitted
		Sharing of results with customers
		Sample acceptation criteria
	Collaborations	Training about antimicrobial resistance
		Scientific publications
		Collaboration with other laboratories in the country
		Collaboration with laboratories outside the country
Technical practices	Resources for bacteriology testing	Biosafety of bacteriology laboratory
		Equipment for bacteriology and AST
		Animal diseases–media and consumable-
		Food safety–media and consumable
		Water and environment–media and consumable
		Plant health–media and consumable
		Reagents availability for AST
	Bacteriology- technical practices	Bacteriology methods
		Bacterial identification
	Antimicrobial susceptibility testing (AST) methods	Standard for AST
		Bacterial inoculum calibration for AST
		Panels definition
		Revision of panels of antibiotics
		Method for reading disk diffusion results
		Method for reading MIC results
		Standard for interpretation of disk diffusion results
		Standard for interpretation of MIC results
	Molecular tools	Molecular characterization (resistance gene confirmation or typing)
		Sequencing of resistant strains
Management of data and biological material	Management of biological material	Sample identification and follow-up
		Proportion of isolates archived in a library
		Method for bacterial preservation
		Inventory of archived isolates
		Duration of bacterial isolates archiving
	Data management	Individual reports on AMR data to the customers
		Data archiving
		AMR data transmission to a dedicated epidemiology unit
Quality assurance	Documentation	SOPs on AMR detection implemented
		SOPs on AMR detection updating
	AMR detection	Reference strains for AST quality control
		Proficiency testing for AST
	Staff	Initial training in AMR testing
		Staff skills validation and continuous proficiency

In both descriptive questionnaires the results are recorded by selecting standardized answers from checkboxes in the Excel^®^ files. For each question, the assessor can provide additional information or comments in a free field box.

In both semi-quantitative questionnaires, the assessors can select a scenario that best describes the situation assessed. Each scenario is related to a score, ranging from 1 (weakest) to 4 (best) per question (Table 3). The Excel^®^ files allow data to be recorded from three different assessments in order to monitor progress over time.

**Table 3:** Example of scoring with the semi-quantitative questionnaires.

**Subcategory**	**4**	**3**	**2**	**1**
Existence of an operational management structure in food and agriculture sectors	A clearly recognized structure exists, its organization is in coherence with the needs of the AMR surveillance system and activities are actively conducted	A clearly recognized structure exists, its organization fits the needs of AMR surveillance but activities are partially conducted	A clearly recognized structure exists but its organization does not fit the needs of AMR surveillance activities OR an epidemiology unit is functional in other fields of food and agriculture (e.g. zoonosis or animal health surveillance) but not involved in AMR surveillance	No dedicated structure OR no structure officially designated for AMR surveillance purpose

In all semi-quantitative questionnaires (LMT-AMR, LMT-BACT and SET-AMR) the assessors can select a scenario that best describes the situation assessed. Each scenario is related to a score, ranging from 1 (weakest) to 4 (best). The example given in that table is taken from the SET-AMR questionnaire, Area: Data production network, Subcategory: Effective integration of competent laboratories in the AMR surveillance system.

A short manual with application guidelines is provided to the assessors directly in the Excel^®^ files, as well as additional information to complete each question. The tool is currently available in four languages (English, French, Portuguese, and Spanish).

## ATLASS assessors and ATLASS community

The ATLASS assessors are either international or national experts with experience in bacteriology and antimicrobial susceptibility testing, and/or applied epidemiology for AMR surveillance in the field of the food and agriculture sectors. The national experts are selected and nominated by countries and attend theoretical and practical training organized by FAO to become ATLASS assessors. This training process ensures standardized assessments from one country to another and from one assessment to another over time. The process to become an ATLASS assessor includes: (1) attending an initial training session (with theoretical lectures and practical exercises) dedicated to the use of the tool as well as the implementation of assessment missions, (2) participation in a mentored FAO-ATLASS mission with an experienced ATLASS assessor and contribution to the drafting of the assessment report, and (3) conducting a FAO-ATLASS mission in autonomy including writing of the report, which is validated according to a defined process. Once completed the three steps, the ATLASS assessor is considered fully trained. Between March 2017 and December 2019, six training sessions have been organized by FAO in Rome (Italy), Singapore (Republic of Singapore), Moscow (Russia), Lusaka (Zambia), Kochi (India) and Dakar (Senegal), gathering 118 trainees from 48 countries.

Besides the short manual available in the Excel^®^ files, the ATLASS assessors are provided an assessor kit that includes generic presentations to be used during the assessment, information material, and guidelines. The guidelines present the structure of the tool, explain how to prepare and conduct assessment missions, and give indications about the expected recommendations, including a standardized report template. They include information about approaches to AMR surveillance and concepts used in FAO-ATLASS. The kit includes a report template to present the assessment results in a standardized manner.

Since March 2017, as an outcome of the training sessions, FAO has been developing and maintaining several ATLASS communities worldwide, enrolling assessors working in government agencies, laboratories, multilateral organizations and academic institutions from different regions/countries in Africa, Asia, and Europe. The ATLASS community serves as regional and national technical resource to monitor and sustain the momentum toward the enhancement of AMR surveillance in the food and agriculture sectors. All ATLASS assessors should regularly conduct FAO-ATLASS assessments, including in their own country, and actively participate in the ATLASS assessors' community in order to ensure a common approach to applying the tool, offer suggestions for possible improvement of the tool and keep up to date with new developments. The participation to the ATLASS community also engages the assessors to participate to information exchange *via* social networking applications or regular coordination/refresher meetings.

In 2019 FAO initiated the training for ATLASS laboratory focal points in each laboratory to ensure familiarity with FAO-ATLASS laboratory module. These ATLASS laboratory focal points are requested to collaborate with the ATLASS assessors during the FAO-ATLASS assessments and to follow up on the recommendations provided to each laboratory. The ATLASS laboratory focal points also complete the regular laboratory self-assessment and share the results with the national ATLASS assessor for the compilation of assessment results from each laboratory within the national network.

## FAO-ATLASS assessment process

The tool was designed to be used through a standardized process either for external assessment of an AMR surveillance system, or by any country as a self-assessment tool when applied by a trained national ATLASS assessor. The suggested approach is to perform an initial external assessment, and then conduct follow up assessments considering the needs of the country either through external or a self-assessment.

Countries can request, on a voluntary basis, an FAO-ATLASS external assessment through their FAO representation. Once the mission is confirmed, a team of two ATLASS assessors is set up with complementary profiles (laboratory and applied epidemiology on AMR). The ATLASS assessors then start to collate information in advance with the assistance of the local FAO office, in particular regarding (i) the main animal (terrestrial or aquatic) and plant production or importation, (ii) the national action plan to combat AMR in the country, the policy and legal frameworks on AMR, (iii) reports and scientific publications about the AMR situation in the country, (iv) results from other previous assessments (e.g., Performance of Veterinary Services (WOAH), Joint External Evaluation (WHO), etc.). Information regarding the national AMR surveillance laboratory network is also necessary to define the laboratories to be assessed in the food and agriculture sector and thus the mission's agenda.

During the assessment mission, which lasts up to 1 week depending on the country situation, an initial briefing meeting is held with all relevant stakeholders involved or to be involved in the national AMR surveillance system. The aim of this meeting is to present the objectives of the mission and gather information on the country's organization through a participatory and multisectoral approach. Although the tool is designed to assess AMR surveillance in the food and agriculture sectors (including environment), key representatives and stakeholders from human health are invited in order to describe and assess the cooperation between all sectors. Additional bilateral meetings may be organized during the week with the main actors of the surveillance system to better detail the information collected during the first meeting, and to cross check information recorded. The information is then recorded using the FAO-ATLASS surveillance module.

During the week, the team visits the selected laboratories which are those included, or intended to be, in the AMR national surveillance system for the food and agriculture sectors (e.g., terrestrial and aquatic animal health, food safety, plant health and environment). Evaluations concern not only each selected laboratory to be assessed but also the functioning of the laboratory network, including the role of the national reference laboratory if existing. These laboratories can either perform antimicrobial susceptibility testing or only provide isolates to be tested by the network, and either be central or district ones. The usual process for each visit includes a first meeting with the laboratory managers to gather information on laboratory's organization and role in the AMR national laboratory network, followed by a technical visit in the bacteriology laboratory. The findings are recorded using the FAO-ATLASS laboratory module (and FAO-ATLASS surveillance module regarding the functionality of the laboratory network).

On the last day of the mission, a restitution meeting is held, ideally with the same stakeholders present on the first day to share information collected during the mission and discuss and agree on a summary of key recommendations. This meeting is also an opportunity to generate a discussion among stakeholders from different sectors about gaps that may be identified in the implementation of the surveillance system in an integrated manner, according to a One Health approach. After the mission, a report is written by the ATLASS assessors, and transmitted to the country for review and clearance. Once cleared, the mission report is officially transmitted to and owned by the country.

## Outputs and recommendations of a national assessment using FAO-ATLASS

The tool can be used to generate a baseline, to monitor progress, and to support countries in building their AMR surveillance system. The results of the assessment are presented in a narrative report that includes figures and tables with semi-quantitative analyses.

Descriptive information addresses the organization of the AMR surveillance system in the country, as well as the capacities of each laboratory visited. A summary of information collected in the descriptive questionnaires allows to automatically generate tables presenting:

- For each laboratory and for the network the activities and AST methods used for each sector,
- The AMR indicators monitored by the country for each sector (type of surveillance, animal species, type of production, sample type, bacterial species, antimicrobial panels).

Besides those specific tables, numerous information can be extracted from the database about the organization of the surveillance (e.g., linkages with AMR surveillance in human health, actors and their roles in the surveillance system), and capacities of the laboratories (equipment, personnel, data management etc.).

**Figure 1:**
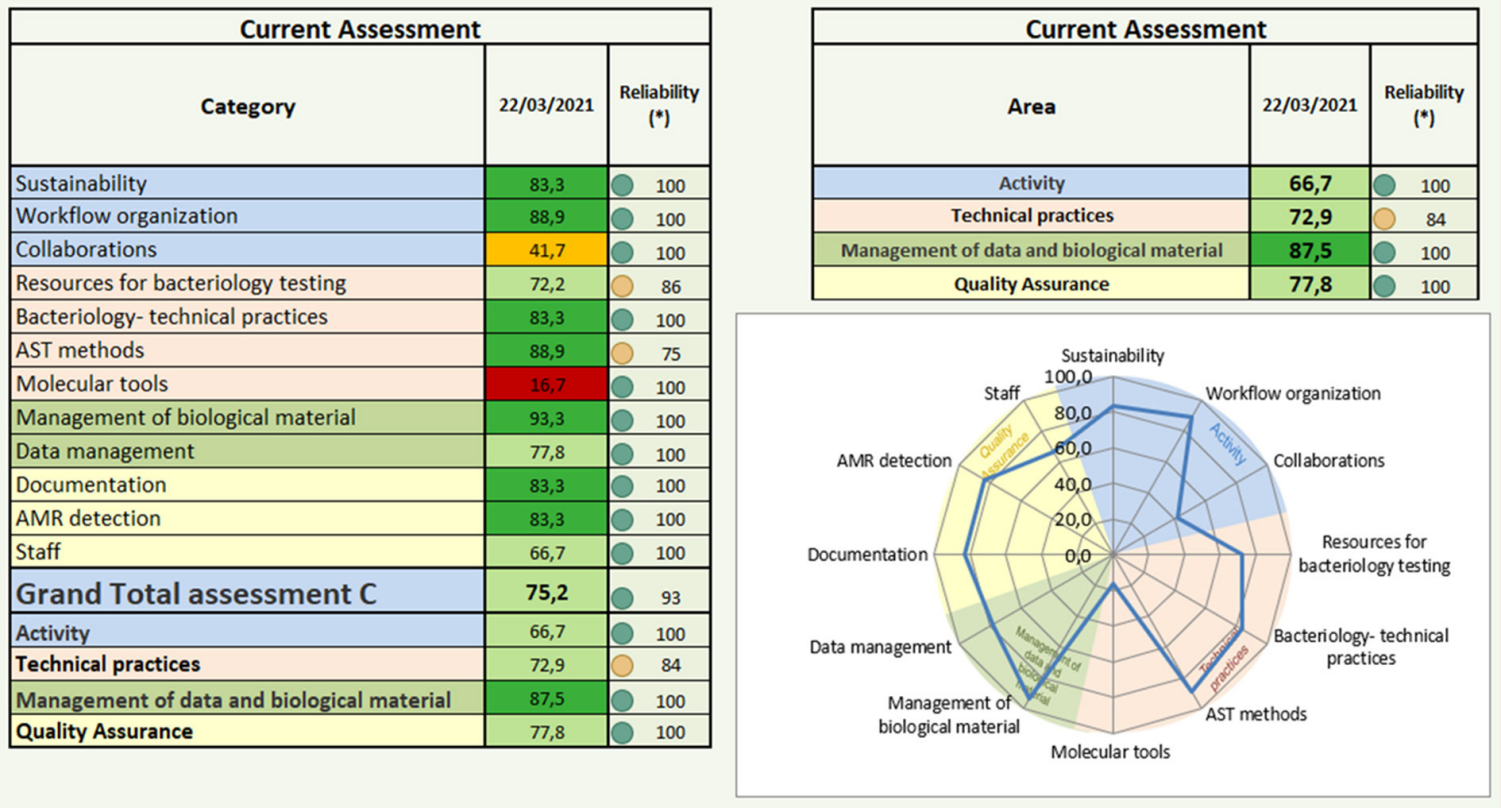
Graphical representation of the laboratory assessment results using the Laboratory Mapping Tool for Antimicrobial Resistance (LMT-AMR). The scoring in this table is based on the ideal situation, with 100% being the score for an ideal laboratory. The number in each cell is the percentage achieved by the laboratory assessed, compared to the ideal. Numbers displayed in percentage; numbers in each cell represent the achieved percentage compared to the optimum (100% being the ideal laboratory). Color coding: 0–20% (dark red), 20–40% (light red), 40–60% (orange), 60–80% (light green, red), 80–100% (dark green). (^*^) Reliability of the result depends on the percentage of questions filled or left blank per category in the LMT questionnaire. From 100 to 90%, the LMT scoring is reliable (green dot). From 90 to 70%, reliability of the scoring is medium (orange dot), from 70 to 0%, reliability is low (red dot).

Quantified results are obtained using the scores (from 1 to 4) from the semi-quantitative questionnaires for each of the laboratories visited and for the surveillance system. The combination of these results allows to:

1) Automatically generate a table and a spider web in the FAO-ATLASS laboratory module to easily summarize strengths and gaps (Figure 1) for each laboratory assessed. LMT-AMR can be also used to compare the results of the current assessment with the two last previous assessments, where such information is available, in order to monitor progress of the laboratory over time.
2) Assign the FAO-ATLASS Progressive Improvement Pathway (PIP) stage ranging from “1-limited” to “5-sustainable” to each of the assessed laboratory and to the surveillance system. The stage “3-developed” is considered as the threshold for claiming reliable activities (data production by laboratories or data use by the surveillance system). As for the Joint External Evaluation (19) scoring process, the laboratory or the surveillance system moves to the next PIP stage only when it has achieved all the attributes of its current PIP. For example, to reach “3-developed” capacity, it has to meet all the attributes of “1-limited” and “2-moderate” stages.

a. For each laboratory, the level of fulfillment of the attributes are expressed as a minimum score to be reached for each question of the LMT-AMR. A summary of the minimum requirements that the laboratory should meet for each specific PIP laboratory stage is presented in Table 4. The same process (using LMT-BACT data) is done for determining the PIP stage of laboratories which role is to conduct bacterial isolation and provide isolates to the network for AMR surveillance purpose.
b. For each area of the AMR surveillance system (governance, data collection and analysis, data production network, communication, and sustainability), the assignment of the PIP stage is based on the fulfillment of essential attributes as assessed by the SET-AMR questions for each area of the surveillance system. An example is given in Table 5 for the PIP stage determination of the “data collection and analysis” area.
c. The overall FAO-ATLASS PIP stage of the national AMR surveillance system is determined by combining the PIP stages of the five main areas. A summary of the minimum requirements that each area of the national AMR surveillance system in the food and agriculture sectors should meet for each FAO-ATLASS PIP surveillance stage is presented in Supplementary Table 1.

**Table 4:** Overview of the main characteristics of a laboratory according to the FAO-ATLASS PIP laboratory stage.

**Stage 1**	**Stage 2**	**Stage 3**	**Stage 4**	**Stage 5**
**Limited**	**Moderate**	**Developed**	**Demonstrated**	**Sustainable**
Very weak workflow organization and financial autonomy No or very weak capacities in AST No or weak quality assurance in the field of bacteriology/AST	Capacity of testing some samples for AST on few pathogens, Weak quality assurance system and/or unstandardized methods for AST and/or gaps in the management of biological material or data	Capacity to test in a standardized manner some samples for AST on few pathogens and to manage biological material and data with basic quality assurance procedures. Challenges may exist for the financial autonomy or the management	Capacity to test in a standardized manner a wide range of bacterial species and to manage biological material and data with robust and sustainable quality assurance procedures AMR data are shared irregularly or partially for surveillance	High-capacity laboratory able to test with a national/international standard a wide range of bacterial species, including fastidious species and to share the results regularly for surveillance or decision making *+ For reference laboratories: able to characterize isolates with molecular tools, and to publish research*

This table summarize the minimum requirements that the laboratory should meet for each of the specific FAO-ATLASS PIP laboratory stages.

**Table 5:** Approach for the determination of the FAO-ATLASS PIP stage of the “data collection and analysis” area.

	**Stage 1**	**Stage 2**	**Stage 3**	**Stage 4**	**Stage 5**
	**Limited**	**Moderate**	**Developed**	**Demonstrated**	**Sustainable**
Existence of an operational management structure (central epidemiology unit) in food and agriculture sectors	≥1	≥2	≥3	≥4	≥4
Frequency of coordination meetings between central epidemiology unit with local units	≥1	≥2	≥3	≥4	≥4
Representativeness of the AMR active surveillance sampling scheme in food and agriculture sectors including environment	≥1	≥1	≥2	≥3	≥4
Representativeness of the sampling of AMR passive surveillance in food and agriculture sectors including environment	≥1	≥2	≥3	≥3	≥4
Adequate skill level in AMR epidemiology of members of the central unit	≥1	≥1	≥3	≥3	≥4
Adequacy of the data management system for the needs of the AMR surveillance system (database, etc.)	≥1	≥2	≥2	≥3	≥4
Data input interval in accordance with the objectives and use of AMR surveillance system results	≥1	≥2	≥2	≥3	≥4
AMR data verification and validation procedures formalized and operational	≥1	≥2	≥2	≥3	≥4
Analysis of AMR data fits the needs of the system	≥1	≥2	≥2	≥3	≥4

The level of fulfillment of the attributes are expressed as a minimum score to be reached for each question of the SET-AMR according to a progressive approach based on each attribute's importance for this area. The same approach is used for the other areas of the surveillance system (governance, data production network, communication, and sustainability). The intensity of the color shades increases with the value of the minimum score to be reached for each question of the SET-AMR questionnaire.

Based on these results the assessors make recommendations adapted to each laboratory, and to each of the five areas, leading to prioritize actions for the improvement of the AMR surveillance system of the country. In that sense, they are presented in a standardized report template making the distinction between first-line priorities which are advised to be implemented within one to two years after publication of the official version of the report, and second line priorities which are advised to be implemented within 3–5 years after publication of the report. The recommendations can address: 1) the governance of the surveillance system related to the national action plan and the AMR coordination committee/working group(s), the strategy for gradual implementation of the national surveillance system, including the identification of possible sampling schemes, the guidance on indicators to be monitored, the modalities of data collection, interpretation, and reporting to authorities and users, etc. and 2) the organization of the laboratory network, including the possible roles and coordination by the National Reference Laboratory, and capacity building required for the laboratories. For each laboratory assessed, a summary table presents the strengths and weaknesses, as well as the recommendations for reaching the next PIP stage.

Missions and workshops (“Post-ATLASS” support) can be organized as a follow up to the ATLASS assessment at country or regional level to gather national authorities and experts to review FAO-ATLASS mission findings, prioritize actions and develop plans for progressive improvement of AMR surveillance. Regional analysis of PIP stages of the surveillance system/laboratories on the countries help to define shared capacity building programs to improve data standardization, such as common AMR indicators and surveillance protocols.

## Discussion

Globally, knowledge of existing AMR surveillance networks in the food and agriculture sector is weak, especially in the low- and middle-income countries (6). The published analysis report of the second round of results of AMR country self-assessment survey showed a sharp contrast between the non-human sectors and the human health sector where most countries have established an AMR surveillance system for common bacterial pathogens (20). On the non-human side, 67 countries (43.5%) collect some data from animal and 60 (38.9%) from food, whereas in the environment and plant sectors most countries have no system in place for surveillance. On the other hand, some countries developed sophisticated surveillance systems, leading sometimes to overlap between national and international systems and the duplication of efforts and economic resources (21). Furthermore, AMR surveillance and monitoring systems vary substantially between sectors and across countries in the type of data collected and reported, as well as laboratory methods. More generally, health care professionals and policy-makers may feel the need to raise awareness of data availability and the potential value of this data, and to ensure that data systems are more accessible (22). Thus, FAO-ATLASS, by mapping and assessing the national AMR surveillance system including all sectors of the food and agriculture and the linkage with AMR surveillance in human health, can be a powerful tool to assist countries in identifying their needs for a robust AMR surveillance system in non-human sectors and thus to make progress in accordance with their AMR national action plans. In that sense, FAO-ATLASS repository allows to share data on the laboratory capacities, as well as the organization and the outputs of the surveillance and plan for harmonized AMR surveillance.

Although some international standards or guidelines on surveillance exist for aspects of food safety and animal health (15–18, 23) significant gaps remain concerning common standards for methods, data-sharing and coordination at local, national, regional and global levels. In that context, the FAO-ATLASS objective is to provide countries a method to assess their AMR surveillance systems in the food and agriculture sectors in a systematic and standardized manner. Assessment data are automatically compiled to assign a Progressive Improvement Pathway (PIP) stage to each laboratory, to each major areas of the AMR surveillance system, and to the overall national AMR surveillance system. The PIP stage is determined on the basis of an internal guideline summarized in Table 4, Supplementary Table 1, which offers countries a progressive development scheme for the organization of the surveillance system and laboratory capacities. This guideline has been reviewed by a multidisciplinary team as part of the revision process and has been adjusted as far as possible with international recommendations. This allows the provision of practical recommendations for laboratory capacity building and surveillance strengthening, which can be prioritized and adapted to the country to ensure a progressive and achievable approach. This also facilitates reaching common and standardized objectives for the implementation of AMR surveillance systems in the food and agriculture sectors worldwide. To assure the standardization of assessments, efforts have also been made to design a formatted and easy to use tool, for the collection and analysis of assessment data. In their surveillance systems evaluation, Calba et al. (24) considered that some of the main limitations of the evaluated approaches were the level of details provided to evaluators for the practical implementation. For FAO-ATLASS we have developed a detailed method with ready-to-use questionnaires designed to assess defined attributes of the AMR surveillance system and produce automatic compilations with graphical representation and scoring. The training process for FAO-ATLASS assessors also contributes to ensure the standardization of the assessments.

Countries around the world are increasingly committed to taking a multisectoral approach to address complex health threats such as AMR at the human-animal-environment interface. As practical implementation of this approach can be challenging, many organizations have provided technical and financial support to countries, using available tools to promote the operationalization of a multisectoral approach. For human medicine, WHO has developed tools to help countries identifying gaps and challenges that relate specifically to participation in the Global Antimicrobial Resistance Surveillance System (GLASS) which fosters standardized AMR surveillance globally, by collecting and reporting data on AMR rates aggregated at national level (25). Some authors developed a roadmap to help low-income countries to participate in this system (26). But although some aspects can be comparable between human and food and agriculture sectors, others, for example sample sources, target organisms, sampling design and laboratory testing, can be quite different. Pelican et al. (27) built a conceptual model representing a consensus on the links and synergies between 12 tools (including FAO-ATLASS, LMT and SET) for advancing One Health implementation, to highlight a potential approach to linking and coordinating the implementation of these tools. In this view, efforts were made to align FAO-ATLASS with other tools, such the Joint External Evaluation (JEE) tool which was developed by WHO for a global multisectoral evaluation process, including the country's capacity to prevent AMR in zoonotic diseases (9). Other tools can be used to describe and evaluate AMR surveillance systems in the food and agriculture and in human health. Recently, some authors provided an overview of what three available tools offer and require from the evaluators, showing that each of them had their strengths and weaknesses in evaluating the different areas and levels of the surveillance systems (28). This study included FAO-Progressive Management Pathway (29), which assesses the progress in the implementation of the country National Action Plan through different focus areas and stages of development for informed decision-making at country level but not meant for comparison between countries. A recent study on the assessment of evaluation tools for integrated surveillance of antimicrobial resistance showed that PMP-AMR and ATLASS seemed to be the most user-friendly tools, particularly designed for risk managers (30). FAO-ATLASS provides deeper insight into the organization of the national AMR surveillance system specifically in the food and agriculture sectors, including assessments of laboratories which are the main data producers for AMR surveillance. Besides the organizational and technical aspects of the AMR surveillance systems, the assessment also concerns governance and funding which are central issues to be considered for assessing the sustainability of a system. Communication and feedback to stakeholders to ensure their awareness and the acceptability of the surveillance system are also taken into account. The semi-quantitative questionnaire developed for the assessment of the surveillance with FAO-ATLASS was based on a previously published tool called OASIS (31) which was also used by FAO as a basis to develop the FAO-Surveillance Evaluation Tool (FAO-SET) which provides countries with a comprehensive and standardized way to evaluate animal disease surveillance systems, including zoonoses. Simultaneously, the semi-quantitative questionnaires developed for the assessment of laboratories involved in the AMR surveillance system were developed on the basis of the FAO-LMT-Core module (32) which can also be used during the assessments to add additional value through describing the functionality and capacity of the laboratory in a more comprehensive way (management of personnel skills, equipment, premises, etc.).

A One Health approach to combat AMR requires the collaboration of multiple sectors. Regarding AMR surveillance, it appears that beyond data integration, the concept of One Health needs to be applied to different tasks, including data collection and analysis, interpretation and dissemination of results (33). FAO, WOAH, UNEP and WHO, also known as the Quadripartite, have a key role in supporting multisectoral responses to AMR. However, there are significant challenges in data sharing and harmonization across sectors to support a One Health response. As reflected in the AMR and AMU surveillance and monitoring information note of the Global Leaders Group on AMR, most data are currently only available in the human health sector and somewhat available in the animal sector, while there is a paucity of data in the plant sector and the environment. More financial resources, more technical capacity, and better infrastructure are needed for AMR/AMU integrated surveillance - particularly in low and middle-income countries. More efforts are needed to use the data generated for informing actions against AMR; and surveillance efforts at all levels, global, regional, and country, need to be coordinated and aligned in data sharing. The Quadripartite organizations are making great efforts to support the generation of information and evidence on AMR and AMU globally. Following the agreement between the organizations to create synergies, WOAH and WHO have established global systems for collecting and analyzing AMU data in terrestrial and aquatic animals and AMR/AMU data in humans, respectively. The collection of data on AMR in animals and food commodities and data on the use of antimicrobial pesticides in crops is under the mandate of FAO and the Organization is currently developing the International FAO Antimicrobial Resistance Monitoring (InFARM) system to cover this information gap in agri-food systems.

Different surveillance approaches and designs can be followed to generate AMR evidence. Building up or strengthening passive laboratory sample-based surveillance, as proposed by GLASS, is a good means to generate AMR data, although it has several limitations in the perspective of clinical decision making, public health practice and epidemiological research, which could be compensated by case-based surveillance (34). In the food and agriculture sectors, active surveillance which involves AMR surveillance in healthy animals entering the food chain, contributes to the pool of information intended to protect human health. Moreover, the availability of standards and possibilities for practical implementation make easier the standardization of surveillance in healthy animals. On the other hand, AMR surveillance in bacterial pathogens from diseased (terrestrial and aquatic) animals also provides a basis for developing evidence-based treatment guidelines that contributes to better antimicrobial stewardship in animals. AMR passive surveillance could also have other advantages: (1) conduct integrated analysis of data obtained under comparable conditions in human and food and agriculture sectors, as surveillance is passive in human medicine; (2) obtaining clinical data to provide the necessary feedback to antimicrobial users in order to improve their practices in the use of antimicrobial; (3) obtaining data for animal species that are harder to reach through active surveillance; (4) strengthening the diagnostic capabilities of bacterial diseases, which is a prerequisite for better use of antibiotics. Standardization and harmonization will allow more meaningful analyses of AMR surveillance under a One Health approach. In that sense, FAO-ATLASS should allow to provide information on the different aspects of the surveillance that should be integrated and ensure the quality and reliability of the data (linked to the PIP stage) used in the surveillance system. FAO-ATLASS will be an essential tool in support of the InFARM system and of the global AMR/U surveillance architecture that is being developed and coordinated by the Quadripartite.

The demand for FAO-ATLASS missions worldwide during the period from 2017 to 2022 demonstrates the interest by FAO member nations in strengthening their capacities for AMR surveillance. Systematic feedback from the FAO-ATLASS community on the tool helps to continuously refine it and address any question associated with interpretation and scoring. Consequently, certain elements of the tool may progressively evolve to take into account this feedback.

## Conclusion

As a global multidisciplinary organization for food and agriculture, FAO plays a key role in providing integrated and coherent support to countries in preventing and minimizing the emergence and spread of AMR across all sectors. Through FAO-ATLASS, FAO provides a valuable tool to help and encourage countries in improving their national AMR surveillance, share reliable AMR data at national level and plan for harmonized regional and global AMR surveillance and data compilations for food and agriculture sectors. The use of FAO-ATLASS is also creating opportunities for laboratory capacity building and increased awareness in countries and regions, which is critical in assuring success in the global fight against AMR under the One Health approach.

## Data availability statement

The original contributions presented in the study are included in the article/Supplementary material, further inquiries can be directed to the corresponding author.

## Author contributions

GD, MT, BM, and NK developed the tool. NK drafted the manuscript which was reviewed by co-authors. EK, OI, and MGor implemented its expansion in Africa (EK and OI) and Asia (MGor). All authors provided their feedback on the tool.
