# Supplementary material for: A systematic approach toward progressive improvement of national antimicrobial resistance surveillance systems in food and agriculture sectors
Source: Front Vet Sci. 2023 Feb 7;9:1057040. doi: 10.3389/fvets.2022.1057040 (PMC9941986; doi:10.3389/fvets.2022.1057040)
Supplement: Supplementary file 1 [file Table_1.docx]

**Table 1. Overview of the main features and characteristics of each area of the national AMR surveillance system according to the FAO-ATLASS PIP surveillance stage**

| AREA OF THE AMR SURVEILLANCE SYSTEM | **Stage 1**  Limited | **Stage 2**  Moderate | **Stage 3**  Developed | **Stage 4**  Demonstrated | **Stage 5**  Sustainable |
| --- | --- | --- | --- | --- | --- |
| **GOVERNANCE** | No national action plan (NAP) for AMR surveillance system and/or no formalized multi-sector collaboration/coordination | NAP under development/not approved or NAP developed with poorly relevant surveillance objectives  Multi-sectoral working group(s) or coordination committee on AMR but activities are not formally defined and/or it meets irregularly | NAP approved and partially implemented with relevant objectives  Multi-sectoral working group(s) including at least human health and animal health sectors with clear terms of reference and regular meetings. Activities, reporting and responsibilities of each member are defined but its composition may be extended | NAP is being implemented and leading to adapted regulations. Relevant sectors involved with a defined monitoring and evaluation process in place, Objectives are fully in compliance with international recommendations  Multi-sectoral working group(s) functional, allowing integrated approaches that include all key stakeholders, used to implement the national AMR action plan | NAP is being implemented and leading to adapted regulations followed by actions. Relevant sectors involved with a defined monitoring and evaluation process in place  Objectives are fully in compliance with international recommendations  Multi-sectoral working group(s) functional, allowing integrated approaches that include all key stakeholders, used to implement the national AMR action plan |
| **DATA COLLECTION AND ANALYSIS (EPIDEMIOLOGY UNIT)** | No dedicated structure or no structure officially designated/competent for AMR surveillance purpose | A dedicated operational epidemiology unit exists, but there are major gaps in the quality of data management or relevance and suitability of AMR surveillance protocols or the epidemiology unit is functional in the field of food and agriculture (eg zoonosis) but not involved in AMR surveillance | A dedicated epidemiology unit is able to collect some AMR data (eg pilot studies, or local data) but may not use a standardized approach and lacks national coordination and/or quality management | An operational epidemiology unit is able to regularly collect and report AMR data in a standardized manner on levels of resistance for at least 2 indicators identified as priority for surveillance, with some minor improvements needed for the sampling scheme and data management | AMR National surveillance system established for indicators identified as priority for surveillance which follows quality assurance processes and with appropriate sampling scheme and strategy. All kind of data are collected in a standardized manner and all of the data are subject to formalized verification and validation procedures |
| **DATA PRODUCTION NETWORK (LABORATORIES)** | No effective integration of competent laboratories in the AMR surveillance system | Some laboratories involved in the AMR surveillance system, but not all, reached FAO-ATLASS PIP stage 3 and/or there are some gaps concerning standardization and data management in the laboratory network | All laboratories involved in the AMR surveillance system reach FAO-ATLASS PIP stage 3 or above, and most of them use the same standard for AST but their role must be better formalized  Minor improvements are needed to improve bacteriology techniques and data management | All laboratories involved in the surveillance system have a clear position, reach a FAO-ATLASS PIP stage ≥3, use the same standard, and adequate bacteriology techniques. The network is coordinated by the National Reference Laboratory (NRL)  Optimal AMR data management in all laboratories but minor improvements needed for data transmission to the epidemiology unit | Robust laboratory network (PIP stage ≥3) using the same international standards and performant bacteriology techniques, coordinated by a National Reference Laboratory (NRL)  Optimal AMR data management in all laboratories involved in the surveillance |
| AREA OF THE AMR SURVEILLANCE SYSTEM | **Stage 1**  Limited | **Stage 2**  Moderate | **Stage 3**  Developed | **Stage 4**  Demonstrated | **Stage 5**  Sustainable |
| **COMMUNICATION** | No communication about AMR surveillance results/risk assessment outcomes / awareness building activity for data sources and/or no identification of key stakeholders’ expectations | No communication about AMR surveillance results/risk assessment outcomes / awareness building activity for data sources and/or no identification of key stakeholders’ expectations | Upstream (e.g. authorities) communication is done on irregular basis and/or information is fragmented  Some key stakeholders may have difficulties to access relevant information | Upstream and downstream (e.g. veterinarians, members of laboratory network) communication of surveillance results/risk assessment outcomes to key stakeholders with some minor improvements regarding the frequency or the possibility to verify that they were effectively disseminated  Key stakeholders’ expectations about the results of the surveillance system to be communicated have been identified and partially taken into account. | Regular upstream and downstream communication through different means (report, newsletter, annual meetings etc.) to all key stakeholders of the AMR surveillance. The modalities of communication are in line with the system’s needs and key stakeholders expectations. Risk assessment outcomes are systematically communicated and followed by specific measures  Key stakeholders’ expectations about the results of the surveillance system to be communicated have been identified and taken into account. |
| **SUSTAINABILITY** | Lack of financial resources is a major constraint for epidemiology or laboratory needs (manpower, equipment and laboratory supplies) | Lack of financial resources is a regular constraint for epidemiology or laboratory needs (manpower, equipment and laboratory supplies).  Performance indicators are developed but improvements are needed. | Financial resources are just enough to fulfil the mandate of the involved laboratories and the epidemiology unit.  Performance indicators are developed but improvements are needed. | NAP and multi-sectoral working group(s) have funding sources identified for the needs of the surveillance system.  Major activities are constrained only slightly by material and financial resources, which may however not allow a timely response to temporary increases in surveillance needs.  Performance indicators are measured but major improvements are needed | NAP and multi-sectoral working group(s) have permanent funding sources which are adequate for the implementation of the surveillance system.  Performance indicators and external evaluations are regularly carried out and corrective measures implemented. |
